# Efficacy of novel regimens targeting oxidative phosphorylation in *Mycobacterium tuberculosis*

**DOI:** 10.1128/aac.00019-25

**Published:** 2025-04-22

**Authors:** Dongshuo Li, Liang Li, Ye Zhang, Kai Cheng, Wenwen Liang, Eric Nuermberger, Xianglong Qi, Lei Fu, Bin Wang, Chenxia Yan, Rui Xu, Yu Lu, Jian Xu

**Affiliations:** 1 Beijing Chest Hospital, Capital Medical University, Beijing Tuberculosis and Thoracic Tumor Research Institute 117550, Beijing, China; 2 Department of Medicine, Center for Tuberculosis Research, Johns Hopkins University School of Medicine 229384 , Baltimore, Maryland, USA; 3 Department of International Health, Johns Hopkins Bloomberg School of Public Health 25802 , Baltimore, Maryland, USA; St George's, University of London, London, United Kingdom

**Keywords:** *Mycobacterium tuberculosis*, oxidative phosphorylation, electron transport chain, bedaquiline, clofazimine, telacebec, pyrazinamide, murine model

## Abstract

*Mycobacterium tuberculosis* in both replicating and non-replicating states relies on oxidative phosphorylation (OxPhos) to generate ATP for its growth and survival. Our research delved into the efficacy of novel regimens targeting the OxPhos pathway in murine models. The combination of bedaquiline, clofazimine, pyrazinamide, alongside telacebec, and SQ109 was investigated against both wild-type *M. tuberculosis* H37Rv and an *Rv0678* mutant with cross-resistance between bedaquiline and clofazimine. The results demonstrated that the combination regimens, particularly bedaquiline + clofazimine + pyrazinamide (BCZ) along with telacebec (BCZT) and SQ109 (BCZS), exhibit significantly enhanced bactericidal activity compared to bedaquiline alone and sterilizing potential against *M. tuberculosis in vivo*. Notably, the BCZT regimen showed superior activity compared to other treatment regimens in *Rv0678* mutant-infected BALB/c mice. The addition of T to BCZ prevented the amplification of bedaquiline-resistant mutants and reduced the number of mice relapsing. Our finding underscores the potential of targeting the OxPhos pathway to combat *M. tuberculosis*, paving the way for innovative approaches in tuberculosis therapy.

## INTRODUCTION

The containment of tuberculosis is imperiled by the emergence and spread of drug-resistant strains, which have reduced the treatment success rate to a mere 60%. It takes up to 24 months for regimens containing at least 4–6 drugs to treat patients with multi-drug resistant *Mycobacterium tuberculosis* (MDR-TB) (1). Novel drug regimens to shorten and simplify TB treatment are urgently needed. *M. tuberculosis* is an obligate aerobic bacterium that depends mainly on oxidative phosphorylation (OxPhos) for energy production via the electron transport chain (ETC) (2, 3). In *M. tuberculosis*, the movement of electrons through a series of ETC complexes, maintenance of the proton motive force (PMF) and synthesis of ATP are essential for growth and survival (2, 4). Even during non-replicating persistence, *M. tuberculosis* uses its ETC to generate the necessary PMF and maintain membrane potential (4, 5), which highlights OxPhos as a promising cell process for drug development.

Recent drug discovery efforts have led to numerous compounds targeting the OxPhos pathway, which have shown great promise in the treatment of tuberculosis. Among these compounds, bedaquiline (B), clofazimine (C), and pyrazinamide (Z) are placed in Group A, B, and C for treatment of MDR-TB by WHO, respectively (6). Telacebec (Q203, T) and SQ109 (S) have been evaluated in phase 2 clinical trials. The diarylquinoline B kills *M. tuberculosis* by disruption of the membrane-bound F1F0-ATP synthase complex, resulting in the depletion of cellular ATP levels (7). The riminophenazine C transfers electrons from type 2 NADH dehydrogenase (NDH2) to O2, leading to the formation of bactericidal reactive oxygen species (ROS) (8). The broad mechanism inhibitor Z and its bioactive metabolite pyrazinoic acid (POA) have been found to decrease the PMF and ATP levels in *M. tuberculosis* (9). The imidazopyridine T inhibits cytochrome bc1 by binding to its QcrB subunit (10), forcing the bacteria to use the less energetically efficient terminal oxidase, cytochrome bd (3). The ethylenediamine S acts as an ETC uncoupler—dissipating the PMF and as an inhibitor of menaquinone biosynthesis at the MenA and MenG steps (11).

The combination of B and C leads to significantly better bactericidal and sterilizing activity in a mouse TB model (12). However, their combined use can lead to selective amplification of cross-resistant mutants with mutations in *Rv0678* and *pepQ* (12). B resistance is conferred by target mutations in the *atpE* gene, as well as non-target mutations in *Rv0678* and *pepQ. Rv0678* mutations are the major mechanism of B resistance among clinical isolates (13–18). Of great concern is the fact that *Rv0678* mutants with reduced B susceptibility have been isolated from TB patients without prior B or C exposure (14, 18, 19). Thus, companion drugs are necessary to restrict the selective amplification of spontaneous B-resistant mutants. The addition of Z to the B-C combination results in even more rapid sterilizing activity in mice (12, 20, 21). The B + C + Z (BCZ) regimen had greater activity than RIF + INH + Z in a murine model of tuberculosis (12, 20–22), where the shortening effect is three or more months (21). B and T kill synergistically with C reportedly by potentiating C’s ROS production (3) and S synergizes with B and C *in vitro* (23).

The development of efficient combination regimens is crucial for shortening anti-TB treatment duration and preventing the occurrence of resistance. Due to the importance of ATP for cellular viability, the combination of drugs targeting elements of the OxPhos pathway can prevent respiratory electron transport, break down the PMF, and block the production of ATP to provide an effective measure against replicating and non-replicating mycobacteria.

In this study, we investigated the efficacy of different regimens targeting OxPhos against infections with a wild-type H37Rv parent strain and an isogenic *Rv0678* loss-of-function mutant in C3HeB/FeJ and BALB/c mice, respectively. BALB/c mice have been widely used to evaluate the effectiveness of tuberculosis drug regimens, as they present with only one single type of cellular, inflammatory lesions following infection with *M. tuberculosis*. C3HeB/FeJ mice develop necrotic granulomas, which may more closely resemble human lesions (1, 24, 25).

## RESULTS

### Bacterial strains and mouse infection model

The schemes indicating the regimens used against *M. tuberculosis* H37Rv and an isogenic mutant with a g193 insertion in the *Rv0678* gene are shown in Table S1 and S2 in the supplemental material, respectively. Using the broth macrodilution method in 7H9 media, the MICs of B, C, S, and T against the H37Rv strain were determined. As shown in Table 1, the B MIC for this *Rv0678* mutant is 0.95 µg/mL (vs 0.06 µg/mL for the parent H37Rv strain) by 7H9 broth dilution at pH 6.8. The MICs of C and T were elevated against the *Rv0678* mutant compared to H37Rv, but the MIC of S did not differ between strains (Table 1).

**TABLE 1:** MICs for B, C, S, and T against H37Rv and *Rv0678* mutant

Strains	MIC (μg/mL)			
	B	C	S	T
H37Rv	0.06	0.12	0.5	0.001
*Rv0678* mutant	0.95	1.82	0.5	0.004

One day after aerosol infection of C3HeB/FeJ mice with H37Rv and BALB/c mice with *Rv0678* mutant, mean (±standard deviation [SD]) lung CFU counts were 2.51 ± 0.13 log_10_ and 2.88 ± 0.31 log_10_, respectively.

### Response to treatment

#### C3HeB/FeJ mice infected with wild-type *M. tuberculosis* H37Rv

As shown in Table 2, by the start of treatment (D0) 6 weeks after infection, the mean CFU count increased to 7.24 ± 0.33 log_10_. Compared to D0, the numbers of CFU in the lungs of untreated mice at 2 weeks (W2) and 4 weeks (W4) into the treatment period were not statistically significantly different (*P* > 0.05). After 2 weeks of treatment, the BCZ regimen reduced the mean lung burden by 3 log_10_ compared to D0 and was more active than B alone and the BCTS regimen (*P* < 0.01). Both the BCZS regimen and the BCZT regimen showed significantly greater killing activity compared to the BCTS regimen (*P* < 0.01) but not compared to the BCZ regimen (*P* > 0.05). After 4 weeks of treatment, CFU reductions of 5.2 log_10_ and 5.5 log_10_ were observed in BCZT-treated and BCZS-treated mice, respectively, compared to untreated mice at D0, and there was no significant difference between the BCZT and BCZS groups. Although the CFU reduction in the BCTS group was smaller than that in the BCZT and BCZS groups (*P* < 0.01), BCTS had greater bactericidal activity than B alone (*P* < 0.05).

**TABLE 2:** Lung CFU counts assessed during treatment against *M. tuberculosis* H37Rv in C3HeB/FeJ mice^*c*^

Regimen^*a*^	Mean lung log_10_CFU count (±SD)^*b*^			
	W6	D0	W2	W4
Untreated	2.51 ± 0.13	7.24 ± 0.33	7.22 ± 0.56	7.34 ± 0.16
B			6.49 ± 0.21	5.75 ± 0.21
BCZ			4.24 ± 0.10	NA
BCZS			4.18 ± 0.29	1.75 ± 0.27
BCZT			4.52 ± 0.23	2.07 ± 0.40
BCTS			6.24 ± 0.25	4.00 ± 0.22

^*a*^ Drug abbreviations are as follows: B = bedaquiline; C = clofazimine; Z = pyrazinamide; T = telacebec; S = SQ109.

^*b*^ Time points are shown as weeks (W6 or W2 or W4) or day (D0) of treatment.

^*c*^ NA, not available due to flood accident.

**Fig 1:**
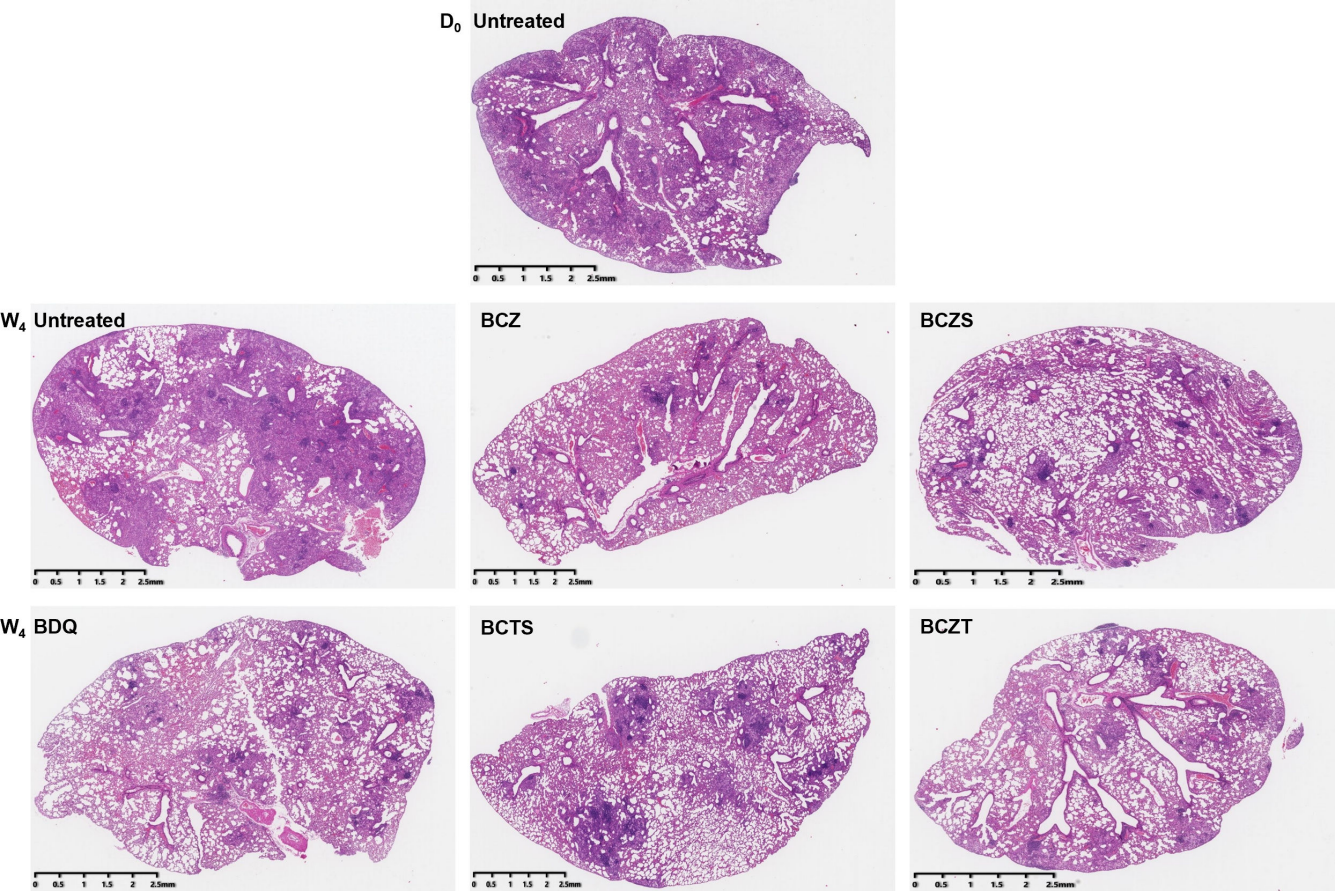
Lung histopathology in C3HeB/FeJ mice before and during treatment with different regimens beginning 6 weeks post-infection with *M. tuberculosis* H37Rv. Hematoxylin and eosin (H&E) staining was performed on lung tissue sections. D0, treatment initiation (6 weeks post-infection); W4, status after 4 weeks of treatment.

At 6 weeks post-infection (D0) with *M. tuberculosis* H37Rv, C3HeB/FeJ mice exhibited extensive lung involvement with granulomatous alveolar pneumonia and pulmonary nodular lesions (Fig. 1). Cellular lesions were composed of neutrophilic clusters interspersed with epithelioid macrophages and lymphocytes. By 10 weeks post-infection (week 4 [W4] of treatment), there was a wide range of nodular lesions in the untreated group, including proliferation of foamy macrophages and granulomatous alveolar pneumonia. There were fewer lung lesions in all B-containing treatment groups compared to the untreated group.

#### BALB/c mice infected with *M. tuberculosis* H37Rv with an *Rv0678* mutation

As shown in Table 3, mean lung CFU counts in untreated BALB/c mice increased at 2 weeks (D0), 6 weeks (M1), and 10 weeks (M2) post-infection to 5.79 ± 0.09, 6.53 ± 0.10, and 7.12 ± 0.11, respectively. As expected, B alone and C alone had no discernible activity against the *Rv0678* mutant after 1 month of treatment (M1) (*P* > 0.05). However, after 2 months of treatment, B alone and C alone each reduced the *Rv0678* mutant mean CFU count to a value approximately 1.8 log_10_ lower than that observed in the untreated group, similar to the effect of B alone against wild-type H37Rv strain after 1 month of treatment in C3HeB/FeJ mice. All tested combinations, including BCZ, BCZS, BCZT, and BCTS, were significantly active against the *Rv0678* mutant, as compared to the untreated group after 1 and 2 months of treatment (*P* < 0.01). The BCZT regimen had the greatest bactericidal activity (*P* < 0.01). Although the BCZS regimen had greater activity than BCTS, BCZS was not significantly different from the BCZ regimen, consistent with W2 results in C3HeB/FeJ mice infected with H37Rv. After 3 months of treatment, although the mean CFU counts were similarly low among BCZ, BCZS, and BCZT groups (*P* > 0.05), two out of five mice in the BCZT regimen were culture negative compared to one out of five mice in BCZ and BCZS regimens (Table 3).

**TABLE 3:** Lung CFU counts assessed during treatment against *M. tuberculosis Rv0678* mutant in BALB/c mice and proportion of mice relapsing after treatment completion

Regimen^*a*^	Mean lung log_10_CFU count (±SD)^*b*^					Proportion of mice relapsing after treatment for:	
	D13	D0	M1	M2	M3	M1 (+3)	M3 (+3)
Untreated	2.88 ± 0.31	5.79 ± 0.09	6.53 ± 0.10	7.12 ± 0.11			
B			6.41 ± 0.35	5.31 ± 0.07			
C			6.31 ± 0.51	5.37 ± 0.10			
BCZ			4.26 ± 0.13	2.05 ± 0.28	0.59 ± 0.37^*c*^	9/9	5/10
BCZS			4.00 ± 0.20	1.88 ± 0.42	0.34 ± 0.41^*c*^	10/10	6/10
BCZT			3.08 ± 0.20	0.62 ± 0.57	0.20 ± 0.30^*c*^	10/10	1/10
BCTS			5.10 ± 0.10	4.60 ± 0.09	4.92 ± 0.04	6/6	10/10

^*a*^ Drug abbreviations are as follows: B = bedaquiline; C = clofazimine; Z = pyrazinamide; T = telacebec; S = SQ109.

^*b*^ Time points are shown as days (D13 or D0) or months (M1, M2, or M3) of treatment. M1 (+3) indicates that the mice were held for three additional months after completing 1 month of treatment.

^*c*^ One out of five mice in BCZ and BCZS regimen was culture negative and two out of five mice in BCZT regimen were culture negative.

**Fig 2:**
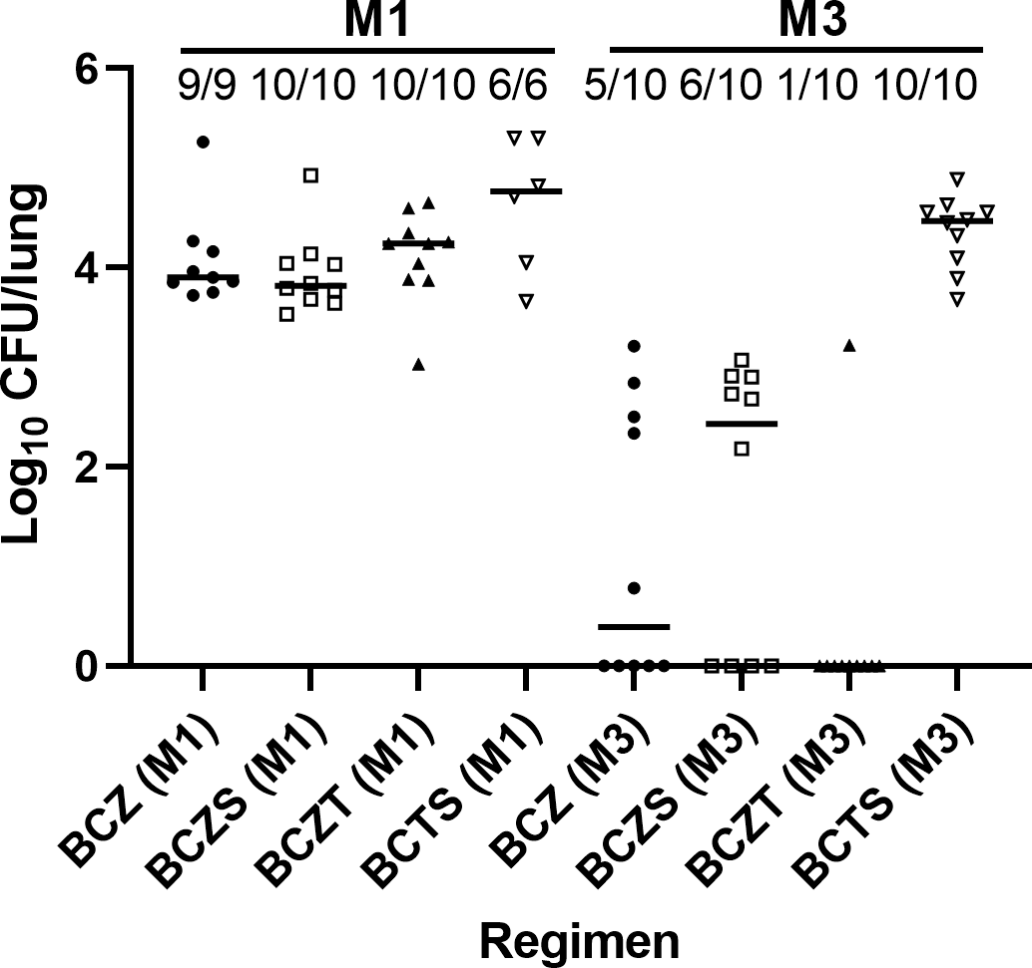
Proportion of BALB/c mice relapsing and individual mouse lung CFU counts after treatment of infection with *M. tuberculosis* H37Rv for 1 month (M1) and 3 months (M3) with each regimen. Regimen symbols: BCZ, solid circles; BCZS, open squares; BCZT, solid triangles, BCTS, open triangles. Horizontal black lines indicate the medians.

Relapse was assessed 3 months after completing 1 and 3 months of treatment in BALB/c mice (Table 3). All mice relapsed after 1 month of treatment with all tested combinations. After 3 months of treatment, the proportion relapsing was lower in BCZT-treated mice (1/10 [10%]) than in BCZS-treated mice (6/10 [60%]). The proportion relapsing of BCZT-treated mice was also lower than that of BCZ-treated mice, but the difference was not statistically significant. The CFU counts at the relapse assessment are shown in Fig. 2.

No mutations were found in *atpE* among the colonies growing on 1 µg/mL B-containing plate from *Rv0678* mutant infected mice at D0, M1, and M1 + 3 (Table S3). No heightened resistance was obtained (Table S3). No growth was observed on 1 µg/mL B-containing plate in any mice after 3 months of treatment with BCZ, BCZS, and BCZT as well as the one relapsing mouse after 3 month of treatment with BCZT. In the mice relapsing after receiving BCZ, BCZS, and BCTS for 3 months, no mutations were found in the *atpE* gene. No new mutations in *Rv0678* were found in all 16 selected colonies on 1 µg/ml B-containing plate.

## DISCUSSION

The present study underscores the significance of targeting the OxPhos pathway in *M. tuberculosis* as a promising strategy for developing new anti-tuberculosis regimens. OxPhos is the central mechanism of energy production for mycobacteria survival and growth (26). *M. tuberculosis* is an obligate aerobe, requiring the ETC for energy production via OxPhos (27). The ETC remains functional in active and dormant *M. tuberculosis* cells (4, 28). Thus, the inhibition of the OxPhos pathway will be an effective strategy to eradicate non-replicating *M. tuberculosis* (29, 30). The mechanisms of action of the drugs in this study are diverse, targeting different components of the ETC and ATP synthesis. This diversity may contribute to the reduced likelihood of resistance development, as the simultaneous disruption of multiple pathways can be more challenging for the bacteria to overcome.

Previous studies demonstrated the enhanced efficacy of the combination BCZ regimen targeting the OxPhos pathway (12, 20). However, their combined use can lead to selective amplification of cross-resistant mutants with mutations in *Rv0678* or *pepQ* (12). Clinical reports of emerging B and C cross-resistance have identified mutations in *Rv0678* that derepress the expression of the MmpL5-MmpS5 efflux transporter as the most common cause (31). It is more problematic that *Rv0678* variants with reduced B susceptibility have been isolated from MDR-TB patients without known prior exposure to B or C (13, 14, 19). These reports raise concern that the emergence of *Rv0678* variants could undermine the clinical efficacy of B-containing regimens. Additional companion drugs are necessary to restrict the selective amplification of B-resistant mutants. Thus, it is important to develop novel regimens targeting OxPhos for suppression of *Rv0678* and to stop further enrichment of target mutations.

The addition of T to the BCZ regimen further potentiated the bactericidal activity in *Rv0678* mutant-infected mice. Among the combined regimens including BCZ, BCZS, BCZT, and BCTS, the BCZT regimen had the best bactericidal activity in *Rv0678* mutant-infected mice. These results are in line with the findings of Lamprecht et al., who reported that a combination of B, T, and C led to a synergistic increase in respiration, ROS, and a depletion of ATP, ultimately resulting in a more lethal effect against *M. tuberculosis* (3). Combined use of OxPhos inhibitors may uncouple the energy metabolism pathway, deplete ATP production, and cause cell death.

The reduced activity of B and C against the *Rv0678* mutant is consistent with previous reports (31) and highlights the need for companion drugs that can bypass or overcome resistance mechanisms. The BCZT regimen demonstrated significant activity against the *Rv0678* mutant, indicating its potential as a treatment option for drug-resistant TB. Moreover, the lower rate of relapse in mice treated with the BCZT regimen for three months suggests that this combination may contribute to a more durable cure. No *atpE* mutations were found from *Rv0678* mutant infected mice that relapsed after treatment with B-containing regimens. This suggests that *Rv0678* mutations may not be a springboard to *atpE* mutations under the conditions tested herein, which is in agreement with a previous study (32).

A limitation of the present study is the absence of pharmacokinetic (PK) studies to assess the plasma exposures of B and C in the treatment regimens. While we did not directly measure the PK profiles of these drugs in the current study, we can reference our previous work for context. In a prior study (20), a single oral dose of 25 mg/kg B in mice resulted in a peak plasma concentration of 1.8 µg/mL, which exceeds the MIC for the *Rv0678* mutant. This suggests that the B dosage used in our regimen is likely to achieve therapeutic levels *in vivo*. It is noteworthy that the plasma concentration of C after a single oral dose of 12.5 mg/kg in mice was 0.24 µg/mL, which is below its MIC for the *Rv0678* mutant (20). Despite this initial concentration, C exhibits accumulation characteristics *in vivo*. Specifically, in our previous study (33), the plasma concentration of C increased from 0.18 to 1.4 µg/mL after 6 weeks of treatment at a dosage of 20 mg/kg in BALB/c mice. This accumulation suggests that the initial plasma concentration may not be indicative of the drug’s presence at later stages of treatment, potentially explaining its observed efficacy despite the initial concentration being below the MIC. Future studies should include comprehensive PK assessments to better understand the drug exposures over time and to guide the optimization of dosing strategies for B and C.

The C3HeB/FeJ pathology was consistent with neutrophil-dominated alveolitis in our study. However, the model is often utilized to study type I necrotic caseating lesions, which were not prominent in this study. Possible explanations for this observation include a high infection dose of 2.5 log_10_CFU; the initiation of treatment at 6 weeks post-infection when lesions may still be in the formative stage; the use of the H37Rv strain, which may be less virulent compared to other strains such as HN878 (1) or Erdman (25). The absence of type I necrotic lesions, which are typically more challenging to treat, represents an additional limitation of our study.

The PRESCIENT trial is currently studying a 12-week regimen of BCZ and delamanid to compare the standard treatment for drug-susceptible pulmonary tuberculosis. In *M. tuberculosis* Erdman-infected BALB/c mice, both the BCZ and BCZS regimens achieved 100% relapse-free cure after only 4 weeks of treatment (22), whereas 60% of mice infected with H37Rv experienced relapse after 4 weeks of BCZ treatment (20). In our study, the relatively high relapse among the BCZ/S and BCTS-treated mice infected with the *Rv0678* mutant is probably due to the elevated MICs of B, C, and T against the *Rv0678* mutant compared to H37Rv. The BCZT regimen had better activity in *Rv0678* mutant infected mice than the BCZ regimen, and the relapsing mice after 3 months of BCZT treatment were lower than those of BCZ-treated mice, which may be attributed to superior drug synergy in BCZT. However, the BCZ regimen was used as a benchmark comparator. Compared to 1 month of standard treatment (INH + RIF + Z, HRZ) regimen with a reduction of 2.2 log_10_CFU, 1 month of BCZ regimen reduced the CFU count by just over 4 log_10_ in H37Rv infected BALB/c mice in a previous study (21). Four months of treatment with the BCZ regimen was as effective as 6 months of treatment with the standard HRZ regimen in preventing relapse in all mice (21). In our study, the significant reduction in lung CFU counts observed after just 2 months of treatment and with a lower proportion of mice undergoing relapse with 3 months of treatment with the BCZT regimen compared to the BCZ regimen suggests that this combination may offer a more rapid path to sterilization in *Rv0678* mutant.

In summary, our study offers a promising outlook for the development of new tuberculosis treatment regimens that target different elements of the OxPhos pathway. The favorable activity of the BCZT regimen over BCZ and its efficacy against B-resistant strains highlights the need for further research and clinical trials to confirm its therapeutic utility.

## MATERIALS AND METHODS

### Bacterial strains and MICs determination

*M. tuberculosis* H37Rv and an isogenic B-resistant mutant with a g193 insertion in *Rv0678* were used in this study. The *Rv0678* mutant was selected from a mouse infected with the H37Rv strain and treated with B monotherapy, and also isolated from spontaneous B resistance mutation experiments (32). The *Rv0678* mutation and the absence of other mutations in genes associated with drug resistance were confirmed by whole-genome sequencing. The MICs of B, C, S, and T against *Rv0678* mutant and H37Rv were determined using the broth macrodilution method (14). Briefly, tubes containing 2.5 mL of 7H9 broth plus 10% oleic acid-albumin-dextrose-catalase without Tween 80 with the twofold dilutions of B concentrations were inoculated with 100 µL containing 2 × 10 CFU of log-phase culture of H37Rv or *Rv0678* mutant, yielding a final testing volume of 200 µL. The MIC was defined as the lowest concentration that prevented visible growth after 14 days of incubation at 37°C.

### Aerosol infection with *M. tuberculosis*

All animal procedures were approved by the Animal Care and Use Committee of Beijing Chest Hospital, Capital Medical University. Female C3HeB/FeJ mice, 10 weeks old, were infected with *M. tuberculosis* H37Rv using the Inhalation Exposure System and a fresh log-phase broth culture, with the intention to implant approximately 300 CFU. Treatment started at 6 weeks after infection (W6). Four mice were sacrificed at W6, and eight mice were sacrificed at D0 for lung CFU counts to determine the number of CFU implanted and the number present at the start of treatment, respectively.

Six-week-old female BALB/c mice were aerosol infected with *M. tuberculosis Rv0678* mutant from a log-phase culture with an optical density at 600 nm (OD_600_) of 0.4, with the goal of implanting 3 log_10_CFU in the lungs of each mouse. Five mice were humanely killed 1 day after infection (D13) and on the day of treatment initiation (D0) to determine the number of bacteria implanted in the lungs and at the start of treatment, respectively.

### Chemotherapy

Drugs were prepared as previously described and administered once daily, 5 days per week, by gavage (31). The drug doses (in mg/kg) were as follows: B, 25; C, 12.5; S, 25; T, 10; and Z, 150. S was suspended in a solution composed of 10% polyethylene glycol (PEG 400) and 90% methylcellulose (0.5%) in distilled water. B, C, T, and Z were dissolved in methylcellulose (0.5%) in distilled water.

### Assessment of treatment efficacy

Efficacy was assessed on the basis of lung CFU counts at selected time points during treatment (a measure of bactericidal activity) and the proportion of mice with culture-positive relapse 3 months after treatment completion (a measure of sterilizing activity). Lung homogenates were plated in serial 10-fold dilutions onto selective 7H10 agar plates supplemented with 0.4% activated charcoal to reduce carryover effects and incubated for 6 weeks before determining final CFU counts for mice on drug treatments.

For relapse assessment, lungs were homogenized in 3 mL of phosphate-buffered saline (PBS), and 2.5 mL of each lung homogenate was plated onto selective 7H10 plates for relapse assessment, at least one of which was supplemented with 0.4% charcoal to control for any possible lingering drug carryover. Positive lung cultures were defined with greater than or equal to 1 CFU of *M. tuberculosis* detected on any plate.

### Pathology

Two mice in each group at each time point were chosen for histopathology. Lungs were fixed in 10% formalin, embedded in paraffin, and cut to 5 µm thickness. Subsequent tissue sections were mounted on glass slides, de-paraffinized, and stained with hematoxylin-eosin (H&E) and Ziehl-Neelsen (AFB) (1). Sections were scanned and visualized using K-Viewer software (KFBIO) for evaluation.

### Evaluation of resistance selection

Aliquots representing one-fifth of the lung homogenates from mice infected with the *Rv0678* mutant were plated directly onto selective 7H10 agar containing 1 µg/mL (16× the wild-type H37Rv MIC) of B to evaluate for *atpE*-mediated resistance at selected time points before, during, and after treatment. Colonies isolated on B-containing plates were selected and analyzed by PCR and DNA sequencing of the *Rv0678* and *atpE* genes.

### Statistical analysis

Lung CFU counts (*x*) were log-transformed (as *x* + 1) before analysis, and mean CFU counts were compared using one-way ANOVA with Bonferroni’s correction to control for multiple comparisons using GraphPad Prism version 6. The proportions of mice relapsing were compared using Fisher’s exact test.

## SUPPLEMENTAL MATERIAL

Supplemental material aac.00019-25-s0001.docx Tables S1 to S3; Fig. S1.

ASM does not own the copyrights to Supplemental Material that may be linked to, or accessed through, an article. The authors have granted ASM a non-exclusive, world-wide license to publish the Supplemental Material files. Please contact the corresponding author directly for reuse.
