# Supplementary material for: Efficacy of novel regimens targeting oxidative phosphorylation in Mycobacterium tuberculosis
Source: Antimicrob Agents Chemother. 2025 Apr 22;69(6):e00019-25. doi: 10.1128/aac.00019-25 (PMC12135524; doi:10.1128/aac.00019-25)
Supplement: Supplemental material — Tables S1 to S3; Fig. S1. [file aac.00019-25-s0001.docx]

Supplementary information

Table S1. Experimental scheme for C3HeB/FeJ mice infected with wild-type *M.tuberculosis* H37Rv

| **Regimen** | **Time point^a^ and No. of mice to sacrifice** | | | |
| --- | --- | --- | --- | --- |
|  | **W-6** | **D0** | **W2** | **W4** |
| Untreated | 4 | 10 | 6 | 10 |
| B |  |  | 6 | 9 |
| BCZ |  |  | 6 | 10 |
| BCZT |  |  | 6 | 10 |
| BCZS |  |  | 6 | 10 |
| BCTS |  |  | 6 | 10 |

aTime points shown as days (D0) or weeks (W-6,W2 or W4) of treatment.

Two mice were used for histopathology at D0, W2 and W4 and the remaining mice were used for the efficacy arm of the study.

Abbreviations: B=Bedaquiline; C=Clofazimine; Z=Pyrazinamide; T= Telacebec; S=SQ109.

Table S2. Experimental scheme for BALB/c mice infected with *Rv0678* mutant

| **Regimen** | **Time point^a^ and No. of mice sacrificed** | | | | | | |
| --- | --- | --- | --- | --- | --- | --- | --- |
|  | **D-13** | **D0** | **M1** | **M2** | **M3** | **M1(+3) ^b^** | **M3(+3)** |
| Untreated | 5 | 5 | 5 | 5 |  |  |  |
| B |  |  | 5 | 5 |  |  |  |
| C |  |  | 5 | 5 |  |  |  |
| BCZ |  |  | 5 | 5 | 5 | 10 | 10 |
| BCZS |  |  | 5 | 5 | 5 | 10 | 10 |
| BCZT |  |  | 5 | 5 | 5 | 10 | 10 |
| BCTS |  |  | 5 | 5 | 5 | 10 | 10 |

^a^Time points shown as days (D-13 or D0) or months (M1, M2 or M3) of treatment.
Abbreviations: B=Bedaquiline; C=Clofazimine; Z=Pyrazinamide; T= Telacebec; S=SQ109.

^b^(+3) indicates mice held for an additional 3 months beyond the completion of treatment (e.g., M1 (+3) indicates mice were treated for 1 months before being held without treatment for an additional 3 months prior to sacrifice).


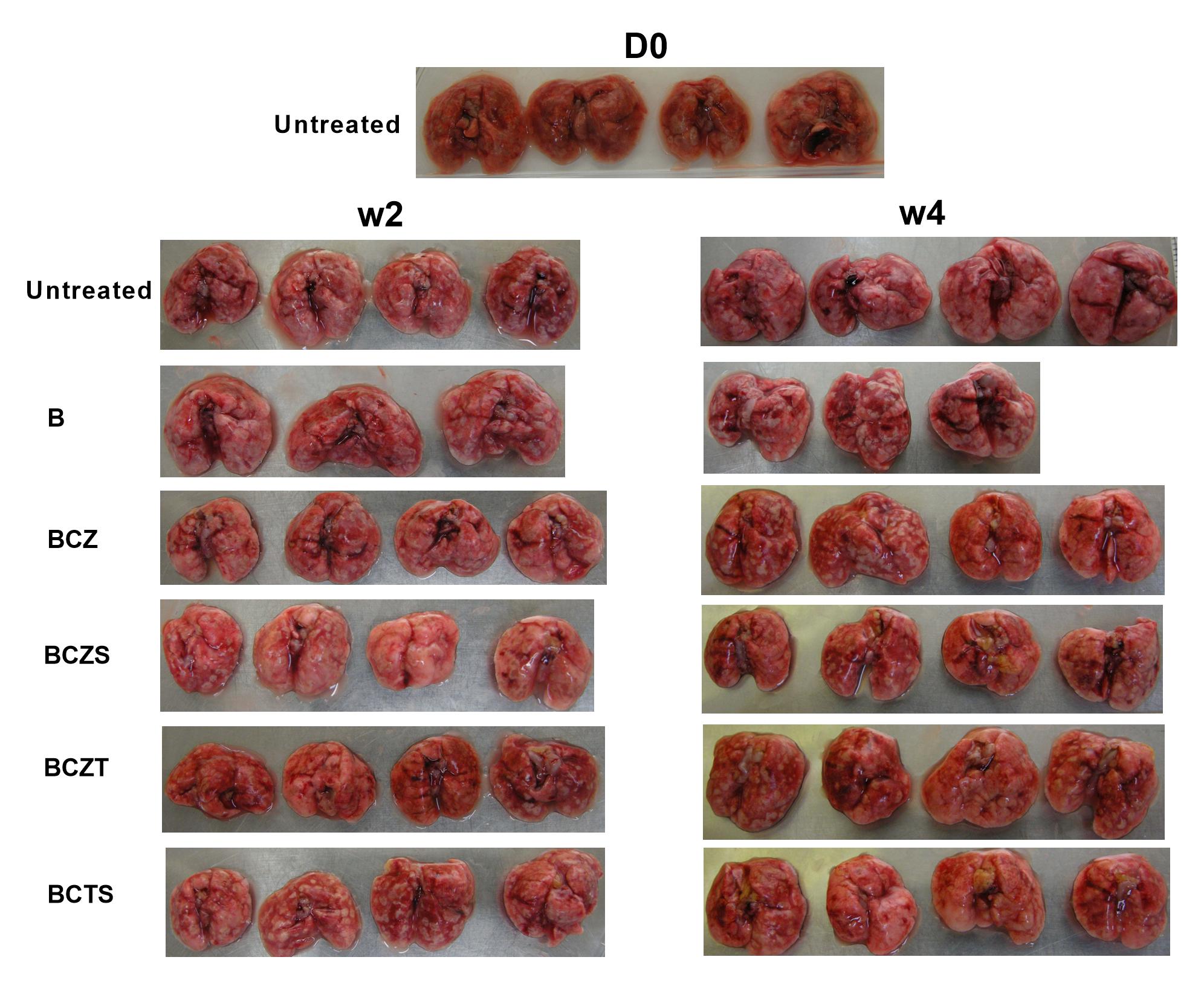


Figure S1. Gross lung pathology observed in C3HeB/FeJ mice before and during treatment with different regimens beginning 6 weeks post-infection with *M.tuberculosis* H37Rv infection.

Table S3 MICs for B and mutations of colonies growing on 1 µg/mL B-containing plate from *Rv0678* mutant infected mice

| Group | B MIC(μg/mL) | *Rv0678* | *atpE* |
| --- | --- | --- | --- |
| Untreated(D0) | 0.95 | G193insertion | WT |
| Untreated(M1) | 0.952 | G193insertion | WT |
| B(M1) | 0.953 | G193insertion | WT |
| C(M1) | 0.960 | G193insertion | WT |
| Untreated(M2) | 0.937 | G193insertion | WT |
| B(M2) | 0.851 | G193insertion | WT |
| C(M2) | 0.953 | G193insertion | WT |
| BCZ(M1+3) | 0.958 | G193insertion | WT |
| BCZS(M1+3) | 0.96 | G193insertion | WT |
| BCZT(M1+3) | 0.954 | G193insertion | WT |
| BCTS(M1+3) | 0.966 | G193insertion | WT |

Note: No test for mutations in the *pepQ* gene.
